# King Edward's Hospital Fund for London

**Published:** 1911-12-16

**Authors:** 


### KING EDWARD'S HOSPITAL FUND FOR LONDON.

A meeting of the Governors and General Council of King Edward's Hospital Fund for London, for the purpose of awarding grants to the hospitals, convalescent homes, and consumption sanatoria, was held on the 11th inst. at St. James's Palace, the Speaker of the House of Commons being in the chair.

Those present were : The Rt. Hon. Sir T. Vezey Strong, Bart., the Duke of Norfolk, Lord Revelstoke, the President of the Hospital Sunday and Hospital Saturday Funds (the Rt. Hon. the Lord Mayor), the Governor of the Bank of England (Mr. A. C. Cole), the President of the Royal College of Surgeons (Mr. R. J. Godlee), the Rt. Hon. Sir Joseph Dimsdale, Bart., the Rt. Hon. Sir Savile Crossley, Bart. (Hon. Secretary), Sir W. S. Church, Bart., Sir Henry Burdett, Sir Horace Marshall, Sir Cooper Perry, Sir John Craggs, Mr. Frederick M. Fry (Hon. Secretary), Dr. Edwin Freshfield, Mr. John G. Griffiths, Mr. Albert G. Sandeman.

In the absence of Lord Rothschild (Honorary Treasurer), Lord Revelstoke reported that the amount received by the Fund for general purposes to December 8, after payment of expenses, was £176,745 0s. 3d.

Sir Henry Burdett stated that the League of Mercy hoped this year that their contribution to the Fund and their distribution amongst the hospitals in the Home Counties would amount together to a sum of £20,000. He could not at the moment say exactly what sum.

### Report of the Distribution Committee.

The Chairman of the Distribution Committee (Sir William Church) read the report of that committee as follows :—

The Governors and General Council having this year placed the sum of £151,000 in the hands of the committee with a view to distribution amongst the London hospitals, as against £150,000 in 1910, the committee beg to report as follows :—

1. The number of hospitals applying for grants is 105, as against 106 last year.

2. Among the institutions which do not appear in this year's list is St. George's Hospital, the Governors of the hospital having withdrawn their application as a result of the decision of the Fund on the question of the amounts which could properly be paid to the Medical School in respect of work done for the hospital. The grant of £2,000 which was reserved last year lapses, the conditions attached to it by the Council of the Fund not having been fulfilled.

3. With regard to the applications received this year from four out of the five throat, nose and ear hospitals, it will be remembered that the Fund has long considered that in the public interest the continuance of five special hospitals for these diseases is not desirable. Various communications upon the subject of amalgamation have passed during the year between one hospital and another, and between different hospitals, jointly as well as individually, and the Fund. No detailed scheme has, however, yet reached the stage at which a grant from the Fund can be recommended. Under the circumstances the committee consider it desirable that the total sums set aside or promised towards a general scheme of amalgamation should be increased to £20,000, and should remain at that figure pending an agreement upon a satisfactory scheme or schemes. The capital grant, promised under certain conditions by the Fund towards a general scheme, amounts to at least £10,000, and the sums now held in reserve in lieu of separate maintenance grants amount to £7,500. The committee therefore recommend that these reserved grants of £7,500 should be added to the capital sum of £10,000, and that a further sum of £2,500 should be added in respect of this year's distribution. The whole amount of £20,000 thus accumulated should be considered as a grant to capital (including building capital), and the committee recommend that they should be empowered to draw upon this sum, in such proportions as they may consider appropriate, in the event of a general or partial amalgamation, as the case may be, being brought to a satisfactory conclusion before the next distribution.

4. None of the grants made to special purposes in 1909 remain unapplied, and no question of the renewal of grants under the two years rule therefore arises.

5. It will be seen from the list of awards that, as in the past, grants have in many cases been made in aid of schemes of improvement or extension which have been submitted to the Fund in accordance with the resolution of the General Council to that effect.

6. The committee are glad to report that the poor district of Kentish Town will shortly be provided with improved hospital accommodation by the rebuilding of the out-patient department of the amalgamated Hampstead and North-West London Hospitals.

7. The sum of £5,000 voted towards the removal of King's College Hospital to South London increases the total amount contributed by the Fund to this object to £42,000. The committee are glad to report that they have received an assurance that every endeavour will be made to push forward the completion of the new hospital and to open it at the earliest possible date.

8. The reports of the Visitors have, as usual, proved to be of the greatest value, and many of their observations have been brought to the notice of the hospital authorities —a practice which has produced useful results in the past.

9. The enlargement of the annual statistical report to cover practically all the hospitals applying for grants has enabled the committee to extend the practice of drawing the attention of the hospitals concerned to cases where the excess of cost above the average seems to call either for specific justification or for greater attention to economy. They desire to record their appreciation of this report prepared by Mr. Maynard which has now at length reached the complete form aimed at from its inception.

### Grants Recommended by the Distribution Committee.

Sir Savile Crossley (Hon. Secretary) presented the list of awards as follows :

Alexandra Hospital for Children.—£300.
Beckenham Cottage Hospital.—£25.
Belgrave Hospital.—£1,500; of which £1,000 to reduce building debt.
Blackheath and Charlton Cottage Hospital.—£150.
Bolingbroke Hospital.—£1,400.
British Lying-in Hospital.—£500.
Central London Ophthalmic Hospital.—£1,500. To rebuilding in accordance with the scheme submitted to the Fund.
Central London Throat and Ear Hospital.—Capital grants amounting to £20,000 have been promised and set aside towards the amalgamation of the Throat, Nose, and Ear Hospitals. See paragraph 3 of Report.
Charing Cross Hospital.—£4,000; of which £1,000 to special appeal for reduction of debt.
Chelsea Hospital for Women.—£500.
Cheyne Hospital for Sick and Incurable Children.—£100. In consideration of the fact that some curable cases are admitted. The Committee are glad to see that a country branch has been opened.
City of London Hospital for Diseases of the Chest, Victoria Park.—£4,000.
City of London Lying-in Hospital.—£1,000; of which £500 to reduce building debt.

Clapham Maternity Hospital.—£100.
Dreadnought Hospital.—£4,500; of which £500 to reduce debt and £500 to underpinning of Docks Branch.
Ealing Cottage Hospital.—£750; of which £500 to rebuilding in accordance with the scheme submitted to the Fund.
East End Mothers' Lying-in Home.—£500; of which £300 to extinguish building debt.
East London Hospital for Children.—£2,000; of which £500 to recent improvements in accordance with the scheme submitted to the Fund.
Eltham and Mottingham Cottage Hospital.—£100.
Evelina Hospital.—
Finchley Cottage Hospital.—£25.
Florence Nightingale Hospital for Gentlewomen.—£250.
French Hospital.—£200.
Friedenheim Hospital.—£50.
General Lying-in Hospital.—£400.
German Hospital.—£200.
Gordon Hospital for Fistula, &c.—£25.
Great Northern Central Hospital.—£4,000; of which £1,000 to reduce debt.
Grosvenor Hospital for Women.—£350.
Guy's Hospital.—£6,500.
Hampstead General Hospital.—£4,500; of which £1,650 on account of maintenance and rent, as agreed upon amalgamation with the North-West London Hospital, and £2,750 to rebuilding of out-patient department in Kentish Town in accordance with scheme submitted to the Fund, on condition that the balance is raised by the Hospital.
Home and Infirmary for Sick Children.—£200.
Home for Mothers and Babies, Woolwich.—£100.
Hospital and Home for Incurable Children, Hampstead.—£150. In consideration of the fact that some curable cases are admitted; of which £100 to lift and isolation ward in accordance with the scheme submitted to the Fund.
Hospital for Consumption, Brompton (including Sanatorium at Frimley).—£4,000.
Hospital for Diseases of the Throat.—Capital grants amounting to £20,000 have been promised and set aside towards the amalgamation of the Throat, Nose, and Ear Hospitals. See paragraph 3 of Report.
Hospital for Epilepsy and Paralysis.—£1,500; of which £1,000 to extension in accordance with the scheme submitted to the Fund.
Hospital for Sick Children.—£3,500; of which £500 to reduce general fund debt.
Hospital for Women, Soho Square.—£1,750; of which £500 to reduction of mortgage on No. 4 Frith Street.
Hospital of St. John and St. Elizabeth.—£400.
Hostel of St. Luke.—£25.
Infants' Hospital.—£50.
Italian Hospital.—£500.
Kensington and Fulham General Hospital.—£25.
Kensington Dispensary and Children's Hospital.—£25.
King's College Hospital.—£7,000; of which £5,000 to removal fund.
Leyton, Walthamstow, and Wanstead Children's and General Hospital.—£300; of which £50 to improvements.
London Fever Hospital.—£100.
London Homœopathic Hospital.—£500.
London Hospital.—£12,000.
London Lock Hospital.—£4,500; of which £500 to the Harrow Road Hospital, and £4,000 to the rebuilding of the Dean Street Hospital in accordance with the scheme submitted to the Fund.
London Temperance Hospital.—£1,500.
London Throat Hospital.—Capital grants amounting to £20,000 have been promised and set aside towards the amalgamation of the Throat, Nose, and Ear Hospitals. See paragraph 3 of Report.
Maternity Charity and District Nurses' Home, Plaistow.—£150.
Medical Mission Hospital, Balaam Street, Plaistow.—£350.
Memorial Hospital, Mildmay Park.—£50.
Metropolitan Hospital.—£3,500.
Middlesex Hospital.—£4,000.
Middlesex Hospital Cancer Charity.—
Mildmay Mission Hospital.—£400.
Miller General Hospital for South-East London.—£2,750; of which £2,000 to extension in accordance with the scheme submitted to the Fund.
Mount Vernon Hospital for Consumption (including Sanatorium at Northwood).—£2,500.
National Dental Hospital.—£450; of which £200 to equipment.
National Hospital for Diseases of the Heart.—
National Hospital for the Paralysed and Epileptic.—£1,750.
New Hospital for Women.—£1,500; of which £750 to structural improvements in accordance with the scheme submitted to the Fund.
Norwood Cottage Hospital.—£50.
Paddington Green Children's Hospital.—£1,250; of which £750 to reconstruction of out-patient department in accordance with the scheme submitted to the Fund.
Passmore Edwards Acton Cottage Hospital.—£75.
Passmore Edwards East Ham Hospital.—£50.
Passmore Edwards Hospital for Willesden.—£50.
Passmore Edwards Cottage Hospital, Wood Green.—£25.
Poplar Hospital.—£750; of which £500 to improvements in accordance with the scheme submitted to the Fund.
Prince of Wales's General Hospital.—£4,000; of which £1,500 to reduce debt.—The Committee consider that this Hospital should receive more support from the public.
Queen Charlotte's Lying-in Hospital.—£750.
Queen's Hospital for Children.—£3,500; of which £1,500 to reduce debt.
Royal Dental Hospital of London.—£750.
Royal Ear Hospital.—Capital grants amounting to £20,000 have been promised and set aside towards the amalgamation of the Throat, Nose, and Ear Hospitals. See paragraph 3 of Report.
Royal Eye Hospital.—£1,000; of which £250 to reduce debt.
Royal Free Hospital.—£3,000.
Royal Hospital for Diseases of the Chest, City Road.—£1,500; of which £250 to reduce debt.
Royal Hospital, Richmond.—£200.
Royal London Ophthalmic Hospital.—£2,250.
Royal National Orthopædic Hospital.—£5,000; of which £3,000 to reduce building debt.
Royal Waterloo Hospital for Children and Women.—£1,750; of which £250 to recent improvements.
Royal Westminster Ophthalmic Hospital.—£100.
St. John's Hospital for Diseases of the Skin, £700; of which £500 to reduce mortgage debt, and £100 to the provision of baths at Leicester Square.
St. John's Hospital, Lewisham.—£350.
St. Luke's House.—£50.
St. Mark's Hospital.—£200.
St. Mary's Hospital.—£5,500; of which £1,500 to reconstruction of Casualty Department in accordance with the scheme submitted to the Fund.
St. Mary's Hospital for Women and Children, Plaistow.—£1,000; of which £250 to roof-garden for patients.
St. Monica's Hospital.—£75.
St. Peter's Hospital for Stone.—£100.
St. Saviour's Hospital, Osnaburgh Street.—£200.
Samaritan Free Hospital.—£1,750; of which £750 to new Nurses' Home in accordance with the scheme submitted to the Fund.
Santa Claus Home.—£25.
South Wimbledon, Merton, and District Cottage Hospital.—£1,000; to the rebuilding of the Hospital in accordance with the scheme submitted to the Fund.
University College Hospital.—£4,000.
Victoria Hospital for Children.—£1,250; of which £250 to recent improvements.
West End Hospital for Diseases of Nervous System.—£350.
West Ham and East London Hospital.—£2,000.
Western Ophthalmic Hospital.
West London Hospital.—£5,000; of which £2,000 to reduce debt.
Westminster Hospital.—£3,500.
Wimbledon Cottage Hospital.—£450; of which £400 to rebuilding in accordance with the scheme submitted to the Fund.

### SPECIAL GRANT.

Set aside for amalgamation of Throat and Ear Hospitals.—£2,500.

### SUMMARY.

| | |
|---|---|
| Grants ... ... ... ... ... ... ... | £148,500 |
| Special Grant set aside for amalgamation ... | 2,500 |
| Total Grants to Hospital for the year 1911 ... ... | £151,000 |

### Report of the Convalescent Homes Committee.

In the absence of Mr. William Latham, K.C. (Chairman of the Convalescent Homes Committee), Dr. Freshfield read the report of that committee as follows:—

1. The Governors and General Council have this year placed the sum of £6,500 in the hands of the committee with a view to distribution amongst convalescent homes and consumption sanatoria. The amount distributed in 1910 was £5,000.

2. The number of applications eligible for consideration amounted to forty-three from convalescent homes and seven from consumption sanatoria, as against forty-five and eight respectively last year.

3. In dealing with applications from convalescent homes the committee have adhered to their original policy of giving special consideration to the convalescent homes closely connected with London hospitals.

4. The committee are glad to report that the scheme for placing beds in country consumption sanatoria at the disposal of patients in London hospitals has proved satisfactory. The hospitals to whom the beds are allotted have all testified to the value of the arrangement and expressed the hope that it will be continued, while the amount of accommodation offered has increased from eighteen beds at three sanatoria to thirty-seven beds at four sanatoria. The accommodation is situated as follows :—

Seven beds at the Sanatorium of the National Association for the Establishment and Maintenance of Sanatoria for Workers, Benenden, Kent.

Eight beds at the Kelling Sanatorium, Holt, Norfolk.

Ten beds at the Devon and Cornwall Sanatorium, South Brent, Devon.

Twelve beds at the Northampton Sanatorium, Creaton, Northants.

5. With regard to the allocation of these beds to hospitals in London the committee have followed the same principle as that adopted last year—viz. that preference should be given (in order of relative size) to the two consumption hospitals which have no country branches, and that the rest of the beds should be divided between the two largest of the general hospitals which apply to the Fund.

They accordingly recommend the following allocation :—

Seventeen beds to the City of London Hospital for Diseases of the Chest.

Eight beds to the Royal Hospital for Diseases of the Chest.

Six beds to the London Hospital.

Six beds to Guy's Hospital.

6. The committee are greatly indebted to the visitors who undertook the task of inspecting the sanatoria applying for grants, and who thus furnished the committee with invaluable evidence as to the suitability of the accommodation provided for London patients by means of the grants made last year.

### A.—Consumption Sanatoria.

The Convalescent Homes Committee desire to draw attention to the fact that the absence of a grant does not necessarily imply dissatisfaction.

Children's Sanatorium, Holt, Norfolk.—£250. To equipment.

Devon and Cornwall Sanatorium, Didworthy, South Brent.—£875. In consideration of the reservation of ten beds during 1912 for the use of certain London Hospitals. (See paragraphs 4 and 5 of Report.)

Eversfield Chest Hospital, St. Leonards.—

Fairlight Sanatorium, Hastings.—

Kelling Sanatorium, Holt, Norfolk.—£700. In consideration of the reservation of eight beds during 1912 for the use of certain London Hospitals. (See paragraphs 4 and 5 of Report.)

National Association for the Establishment and Maintenance of Sanatoria for Workers, Benenden, Kent.—£625. In consideration of the reservation of seven beds during 1912 for the use of certain London Hospitals. (See paragraphs 4 and 5 of Report.)

Northampton Sanatorium, Creaton, Northants.—£1,050.—In consideration of the reservation of twelve beds during 1912 for the use of certain London Hospitals. (See paragraphs 4 and 5 of Report.)

Total ... ... ... £3,500.

### Grants Recommended by the Convalescent Homes Committee.

Mr. Frederick M. Fry (Hon. Secretary) presented the list of awards as follows :—

Alexandra Hospital for Children, Painswick.—£25.

All Saints' Convalescent Home for Children, Highgate.—£25.

Baldwin Brown Home for Convalescent Poor, Herne Bay.—£25.

Brentwood Convalescent Home for London Children, Brentwood.—£25.

Catherine Gladstone Free Convalescent Home for the Poor, Mitcham.—£50.

Charing Cross Hospital, Limpsfield.—£100.

Chelsea Hospital for Women, St. Leonards.—£50. With a view to encouraging the admission without payment of patients direct from the Hospital.

Children's Cottage Hospital, Cold Ash, Newbury.—£25. In consideration of convalescent cases.

Children's Home Hospital, Barnet.—£25. In consideration of convalescent cases.

Convalescent Police Seaside Home, Hove.—£25.

Deptford Medical Mission, Bexhill. £25.

East London Hospital for Children, Bognor.—£250.

Erskine House, Hampstead Heath.—£50; of which £25 to reduce debt.

Great Northern Central Hospital, Clacton-on-Sea.—£150.

Home and Infirmary for Sick Children, Sydenham, Broadstairs.—£50.

Hospital for Sick Children, Great Ormond Street, Highgate. £200.

King's College Hospital, Hemel Hempstead.—£100.

London Hospital, Tankerton.—£75.

Mary Wardell Convalescent Home for Scarlet Fever, Stanmore.—£25.

Metropolitan Convalescent Institution (Children), Broadstairs.—£100.

Metropolitan Convalescent Institution, Walton.—£100.

Middlesex Hospital, Clacton.—£400.

Mildmay Mission Hospital, Hendon.—£100

Mrs. Kitto's Free Convalescent Home, Reigate.—£75.

National Hospital for the Paralysed, Finchley.—£100. With a view to facilitating the admission of patients without payment.

Paddington Green Children's Hospital, Slough.—£100.

Poplar Hospital, Walton-on-Naze.—£50.

Queen Charlotte's Lying-in Hospital, Kilburn.—£50.

Queen's Hospital for Children, Bexhill.—£100; of which £50 to equipment.

St. Andrew's Convalescent Home, Folkestone.—£50.

St. Andrew's Convalescent Hospital, Clewer.—£25.

St. Mary's Home for Children, Broadstairs.—£75. In consideration of convalescent cases.

St. Michael's Home for Men and Women, Westgate-on-Sea. —£25.

Sunshine Home of Recovery, Hurstpierpoint.—£25.

Surgical Home for Boys, Banstead.—£25.

Victoria Home for Invalid Children, Margate.—£50. To Building, in consideration of convalescent cases.

Victoria Hospital for Children (Chelsea), Broadstairs.—£225.

Winifred House Invalid Children's Convalescent Hospital Home, Tollington Park.—£25.

| | |
|---|---|
| Total ... ... ... ... ... ... ... | £3,000 |
| Grants to Consumption Sanatoria ... ... ... | £3,500 |
| Grants to Convalescent Homes ... ... ... ... | £3,000 |
| Total Grants recommended ... ... ... ... | £6,500 |

The Speaker of the House of Commons, in moving the adoption of the reports and awards, said : My Lords and gentlemen,—It falls to my lot this year to preside at the distribution meeting, and to propose the motion which stood in the name of the Duke of Teck last year, and for so many years was moved by his present Majesty, when Prince of Wales and President of the Fund—namely, the adoption of the reports of the Distribution and Convalescent Homes Committees.

The absence of my colleagues, the Duke of Teck and Lord Iveagh, who are both attending the ceremonies connected with his Majesty's visit to India, has rendered it necessary to put into operation the clause of the Act of Incorporation which authorises the Governors to delegate their powers to a committee. They have, therefore, with the approval of his Majesty, the Patron of the Fund, appointed Lord Donoughmore and the Rt. Hon. Sir

Thomas Vezey Strong to exercise with myself the powers of the Governors for the time being.

Message from the King.

Before proceeding to the business of the motion before us, I have to communicate to the Council the following message which we have had the honour of receiving from his Majesty :—

"Emperor's Camp,
"India,
"9th December, 1911, 2.45 p.m.

"On the occasion of the annual meeting on Monday I wish to congratulate the Governors and Council of the Fund upon the satisfactory results achieved this year. I am glad to think that they can increase the annual distribution by £2,500, indicating the continued support of the public and confidence in its efficient administration. I highly appreciate the generous and untiring work given to the Fund by the Honorary Secretaries and members of the various committees, and I take as ever the deepest interest in its welfare.

"George R.I."

I feel sure that I shall be voicing the sentiments of all present here to-day if I express our deep appreciation of his Majesty's kind message and our thanks to him for his remembrance of our meeting and work, in the midst of the absorbing and moving scenes with which his Majesty's attention is now so fully occupied. (Hear, hear.)

Steady Progress.

The first point that arises from a consideration of the reports which are before us is the steady progress (referred to in his Majesty's gracious message) in the amount distributed. The total this year is £157,500, representing an increase of £2,500—£1,000 in the amount distributed to hospitals and £1,500 in the amount distributed to convalescent homes and consumption sanatoria.

Among the receipts which have made possible this progress I would mention first the increased annual subscription of 100 guineas with which her Majesty the Queen has honoured the Fund. This, following as it does upon the increase of last year of his Majesty's subscription from £500 to £1,000, and the subscription of £100 from H.R.H. the Prince of Wales, is yet another mark of the interest taken by the Royal Family in the Fund, and their recognition of the needs of the hospitals of London. His Majesty has also allocated to the Fund a share amounting to £2,000 in the munificent Coronation gift of his Highness the Maharaja of Gwalior.

The receipts again include an anonymous donation of £10,000; the City Corporation have for the third time in succession voted an annual subscription of £500 for five years; while several of the City Companies have continued their welcome donations to the Fund. The contribution of the League of Mercy continues to be one of the largest items in the list of receipts.

Withdrawals from List of Applicants.

The Council will have observed the withdrawal of St. George's Hospital from the list of applicants. It is a matter of deep regret to all of us that any difficulty should arise between the Fund and any of the hospitals. But the Fund is bound by the findings of Sir Edward Fry's Commission on the Financial Relations between Hospitals and Medical Schools, and it has no option but to observe them both in the letter and in the spirit.

The Uniform System.

In the same way the Fund is bound by the rules of the Revised Uniform System of Hospital Accounts. This system, it will be remembered, was adopted by the three Metropolitan Funds, after full discussion with a committee of hospital secretaries, not as being the only form in which the accounts of hospitals could legitimately be kept, but as being a sound and practicable method of securing the commonly admitted benefits of a general uniformity. As such it has become the recognised standard of hospital account-keeping; intending subscribers rely upon the system as a means of comparing one hospital with another, and in fairness therefore both to the public and to the hospitals generally it is necessary to see that its rules are observed by all institutions applying for grants. In this connection a word of thanks is due to the accountants and auditors who have carried out the system, and whose professional services are so often given to charitable institutions either free or for merely nominal remuneration.

Grants to Special Purposes.

The Council will notice that grants to special purposes again form an important feature in the distribution. It must not be supposed, however, that the thorough consideration by the Distribution Committee of schemes submitted to the Fund has only taken place where special grants appear in the list. It often happens that the committee pass a scheme and yet do not consider that the circumstances demand the ear-marking of any portion of the Fund's grant. In recommending all awards, whether allocated to a special purpose or not, the committee take into consideration all the facts which come to the knowledge of the Fund, including those submitted by the hospitals themselves, or contained in the visitors' reports, as well as those revealed by the published accounts and balance-sheets and statistics of cost of working.

The Year's Work.

The Council will have been pleased to hear of the success of the scheme of the Convalescent Homes Committee for placing beds in consumption sanatoria at the disposal of London Hospitals, and of the increase in the number of beds secured in this way for next year.

In July last the Executive Committee issued a circular to the principal hospitals most likely to be concerned, inviting expressions of opinion as to the probable effect of the National Insurance Bill upon their finances and the numbers of their patients. A short statement summarising the general effect of the replies was prepared by the committee and was published in the Press.

Lamented Deaths.

It is with deep regret that the Governors have to record the death of Lord Northcote, who at the time of his illness was giving the Fund the benefit of his invaluable assistance as a member of the Special Committee which, under the chairmanship of Lord Mersey, has been inquiring into the Out-patient system. In spite of the delays resulting from the loss of the services of their colleague, the committee had before the end of the summer nearly completed the receipt of evidence. They did not, however, feel that it was desirable to settle their report while the National Insurance Bill was before Parliament, and still liable to changes which might affect its relation to the subject of their inquiry.

Besides the death of Lord Northcote, the Council has also to lament the loss of two of its original members, the Rev. Dr. Adler (the Chief Rabbi) and the Rev. J. Guinness Rogers, while the Fund has lost a useful visitor by the death of Dr. Rainy.

Thanks to the Workers.

To all the members of the Council and committees and to the visitors and the honorary officers the Governors desire to tender their best thanks. It is with great regret that we have learned that Mr. Fry is unable through pressure of other work to continue his invaluable services in the responsible position of one of our honorary secre-

(*Continued on page* 294).

taries. It would be difficult to say in a few words how much the Fund has owed during the last four years to his great experience in administrative and charitable work, and to his untiring devotion to the performance of any duty which he has taken up. (Hear, hear.) It is some consolation to know that we shall still retain his services on the Council and on the Distribution Committee. The Governors, with the approval of his Majesty the King, Patron of the Fund, have invited Mr. John G. Griffiths to take his place as one of the honorary secretaries and as a colleague to Sir Savile Crossley. Mr. Griffiths has been associated with the Fund for many years, and he is well known in the hospital world through his work in connection with the Revised Uniform System of Hospital Accounts.

I have now, my Lords and gentlemen, to move the adoption of the reports.

The Duke of Norfolk having seconded the motion, the adoption of the reports was then put and carried unanimously.

On the motion of Sir Joseph Dimsdale, seconded by Dr. Freshfield, it was resolved that the cheques for the grants should be posted on December 19, except in the case of grants for the reservation of beds in consumption sanatoria during 1912, which should be posted on January 2.

### Arrangements for 1912.

The Speaker of the House of Commons then said : My Lords and gentlemen,—In accordance with the custom of the Fund, I have now to announce the intentions of the Governors with regard to the appointment of the General Council and Committees for the year 1912. Briefly our present intention is to reappoint the whole of the existing members both of the Council and of the committees, including, of course, the Out-patient Inquiry Committee, whose work is not yet finished.

It only remains to consider the delegation resolutions which have been circulated with the agenda, and then, if they are approved, the meeting which will be held in the New Year to pass them need only be formal.

On the motion of Sir Horace Marshall, seconded by Mr. Albert G. Sandeman, a resolution was approved delegating powers to the Finance, Distribution, Convalescent Homes, and Out-patient Inquiry Committees.

On the motion of Sir Cooper Perry, seconded by Sir John Craggs, a resolution was approved delegating powers to the Executive Committee.

The Speaker of the House of Commons then moved the following resolution, which was seconded by the President of the Royal College of Surgeons (Mr. R. J. Godlee), and carried : "That the keys of the lock under which the Corporate Seal is kept be held as follows : One of the three keys comprising one triplicate set shall be held respectively by the Earl of Bessborough, the Lord Rothschild, and Mr. John G. Griffiths, and one of the three keys comprising the other triplicate set shall be held respectively by the Lord Revelstoke, the Rt. Hon. Sir Savile Crossley, Bart., and Mr. Frederick M. Fry."

On the motion of Sir Henry Burdett, seconded by Sir Savile Crossley, a resolution was passed expressing the thanks of the Council to Mr. Fry for his services as Hon. Secretary.

The Lord Mayor (Sir Thomas Crosby) proposed a vote of thanks to the Speaker of the House of Commons for presiding. The resolution was seconded by the Governor of the Bank of England (Mr. A. C. Cole), and carried unanimously.

The Speaker having briefly acknowledged the vote of thanks, the proceedings then terminated.

